# When to Use Antibiotics in COVID-19: A Proposal Based on Questions

**DOI:** 10.7759/cureus.27398

**Published:** 2022-07-28

**Authors:** Carmelo Dueñas-Castell, Camilo Jose Polanco-Guerra, Maria Cristina Martinez-Ávila, Amilkar J Almanza Hurtado, Tómas Rodriguez Yanez, Juan Camilo Gutierrez-Ariza, Jorge Rico-Fontalvo

**Affiliations:** 1 Intensive Care Medicine, Gestión Salud IPS, Cartagena, COL; 2 Internal Medicine, Universidad del Sinú - Elías Bechara Zainúm, Cartagena, COL; 3 Intensive Care Medicine, Nuevo Hospital de Bocagrande, Cartagena, COL; 4 Intensive Care Medicine, Universidad de Cartagena, Cartagena, COL; 5 Critical Care Medicine, Universidad de Cartagena, Cartagena, COL; 6 Critical Care Medicine, Clinica La Ermita, Cartagena, COL; 7 Nephrology, La Asociación Colombiana de Nefrología e Hipertensión Arterial, Bogotá, COL

**Keywords:** bacterial coinfection in covid-19, superinfection, antibiotics therapy, coronavirus disease 2019 (covid-19), sars-cov-2 (severe acute respiratory syndrome coronavirus -2)

## Abstract

The COVID-19 pandemic has affected millions of people, including hundreds of deaths. The search for adequate treatments and interventions that influence poor prognostic factors and reduce mortality has led to excessive use of antibiotics based on the possible existence of bacterial co-infection. However, there is no evidence to justify the systematic use of antimicrobials in COVID-19. The recommendations seek to provide knowledge regarding treatment; standardizing a management algorithm requires validation in clinical trials and studies of greater methodological rigor.

## Introduction and background

Even though COVID-19 is a viral disease, the frequent use of antibacterial agents for its treatment is a concern [1]. During the initial stages of the pandemic, there was an indiscriminate use of these agents based on the fact that viral respiratory infections predispose to bacterial superinfection [1]. A meta-analysis established the presence of bacterial co-infection in COVID-19 at 7%, increasing to 14% in the population of the ICU [2]. These results are far from those presented in other viral infections such as influenza A H1N1, where bacterial co-infection was estimated in 30% of cases, behaving as an independent mortality factor [3]. Furthermore, these infections could negatively impact innate and adaptive immunity since they surpass the weakened immunological barrier, causing fatal clinical complications [1]. It is worth mentioning that respiratory failure from COVID-19 pneumonia is the commonest cause of death [4]. 

The clinical spectrum of COVID-19 varies from paucisymptomatic forms to critical illnesses that may initially mimic a common cold. On the other hand, imaging modalities often show abnormalities in the more advanced stages of infection [1,5,6]. Therefore, differentiating COVID-19 from other infectious states (bacterial or viral) is difficult [1,5,6]. In short, in our midst, limited resources make it even more challenging to distinguish between COVID-19 and bacterial superinfection, or co-infection. All these factors present a challenge when applying a policy of rational use of antibiotics [1,5,6]. This review aims to describe the available evidence on antimicrobial management of COVID-19 and propose a strategy for their appropriate use.

## Review

How does the infection occurs?

**Figure 1:**
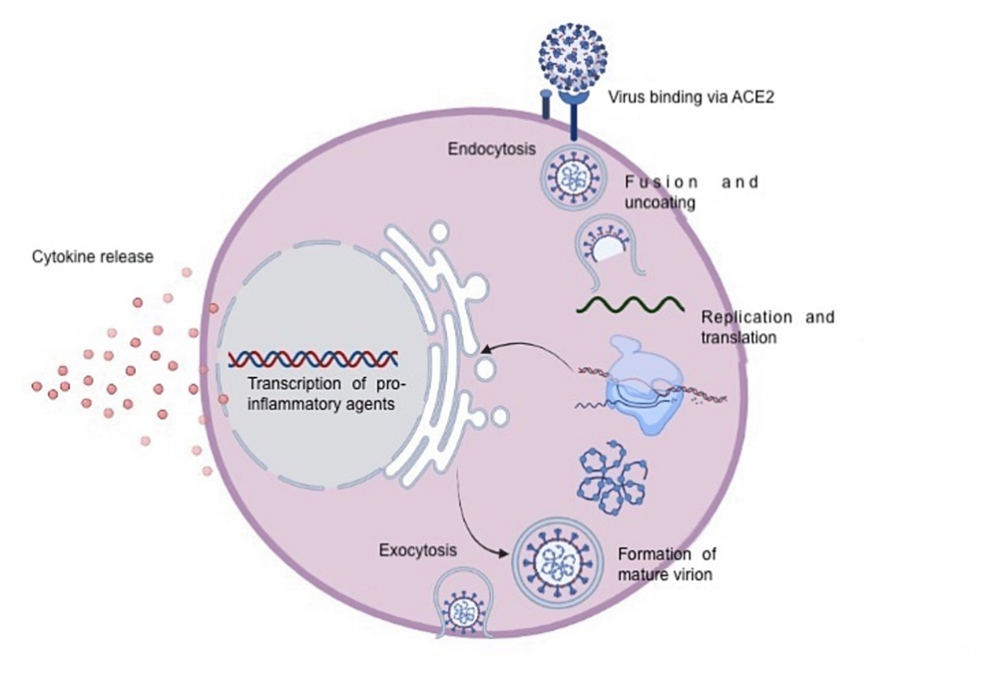
SARS-CoV-2 replication cycle.

Several phases in the development of COVID-19 have been described [7,8]. SARS-CoV-2, the etiological agent, binds to cells through the ACE-2 receptor and, after endocytosis, fuses with endosomes, initiating stage I or the viral phase. Subsequently, the virus releases its RNA intracellularly, and the host cell organelles are used to replicate the peptide chains and produce new viruses [8]. Finally, SARS-CoV-2 virions are released from the host cell by exocytosis and invade nearby cells (Figure 1) [9].

**Figure 2:**
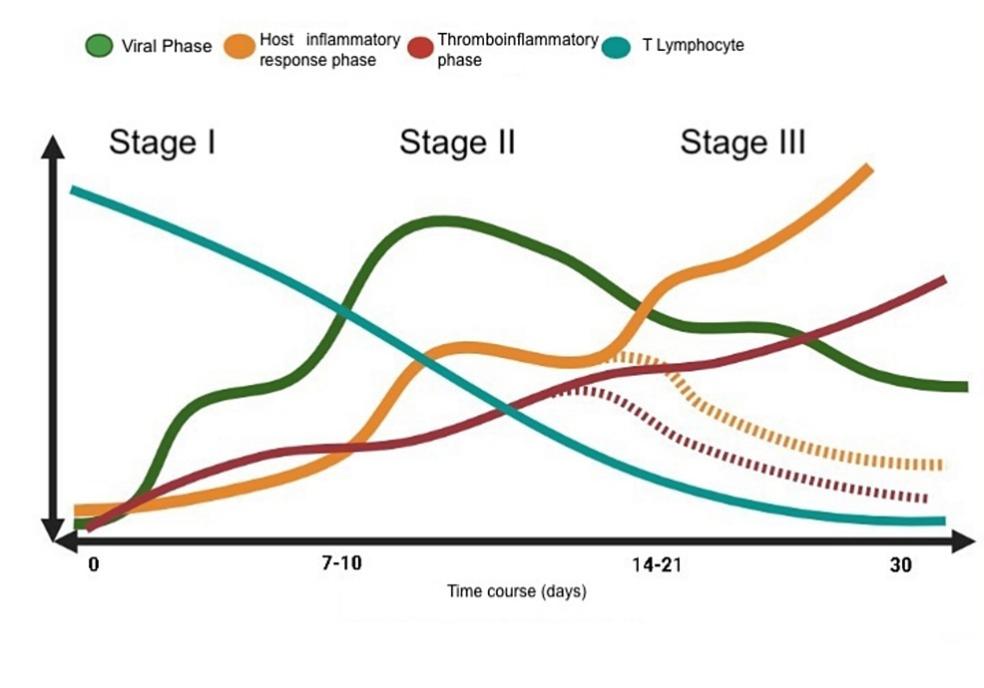
Stages of SARS-CoV-2 infection. Green line: Viral phase; Yellow line: Host inflammatory response phase; Red line: Thromboinflammatory phase; Blue line: T-lymphocyte concentrations; Flashing lines: Progression to a resolution phase.

After respiratory and alveolar dissemination, stage II (symptomatic) begins. This stage is also called the acute or critical phase. As it progresses through the bloodstream, the virus reaches other organs and systems [10]. By invading epithelial cells, SARS-CoV-2 stimulates an immune-mediated inflammatory response, giving rise to stage III ( hyperinflammatory), where tissue damage, endothelial dysfunction, and exaggerated release of cytokines occur, namely IFN-γ, TNF-α, IL -1, IL-10, IL-7, IL-2, IL-6 [11]. This leads to the activation of procoagulant factors, increased permeability, inflammation, vascular dilation, along with intravascular coagulation, ischemia, thrombosis, pulmonary edema, respiratory failure, and multiple organ failure [12-15]. During phase II or III, the immune response, amount of cytokines, and inflammatory mediators can be modulated, achieving effective control of the virus, clinical improvement, and increased number and functionality of T lymphocytes; this is called the recovery phase (Figure 2) [7,8].

What do we use to treat COVID-19?

Early in the pandemic, there were no specific therapeutic interventions. Initially, antiviral drugs such as lopinavir/ritonavir, remdesivir; interferons; convalescent plasma; antimalarials (chloroquine or hydroxychloroquine); and ivermectin were used. Nevertheless, today there is solid evidence against its use, even showing deleterious effects [16]. Therefore, in addition to symptomatic treatment, different options have been proposed for the most critical phases of the disease [17,18].

Recently, targeted antiviral therapy (molnupiravir, paxlovid) and anti-SARS-CoV-2 monoclonal antibodies (bamlanivimab/etesevimab, casirivimab/imdevimab) have been approved under FDA issued Emergency Use Authorization (EUA) [19]. However, they do not decrease mortality.

The Randomised Evaluation of COVID-19 Therapy (RECOVERY) study showed that when using dexamethasone, there was a notable decrease in 28-day mortality in critically ill patients with COVID-19 and a lower oxygen requirement, especially invasive mechanical ventilation (IMV) [20]. Furthermore, a retrospective cohort found that the likelihood of developing secondary infection was not significantly affected by corticosteroid administration (HR: 1.45, CI: 0.75-2.82, p=0.28), and this was held constant in the sub-analysis examining blood, urine, and sputum cultures to prove any sign of bacterial infection; concluding that the diagnosis of bacterial superinfection did not have a significant impact on the probability of mortality in 28 days (HR: 0.66, CI: 0.33-1.35, p=0.25) [21].

Another endorsed drug is tocilizumab, an IL-6 inhibitor, which reduces mortality and IMV requirements without significantly increasing serious adverse events [22]. However, a single-center cohort found that the use of tocilizumab was associated with a higher proportion of bacterial superinfection (54% vs. 26%; p<0.001); no difference in the 28-day case fatality rate among tocilizumab-treated patients who developed bacterial superinfection (22% vs. 15%; p=0.42) [22].

Antibiotics/Antimicrobial resistance: a latent threat

Antibiotics do not directly affect SARS-CoV-2, but viral respiratory infections predispose to bacterial pneumonia [1]. Although, the use of antibiotics with antiviral properties has been proposed [18], macrolides such as azithromycin have antiviral actions in addition to anti-inflammatory actions by attenuating the immune response, effects ruled out by current evidence [23, 24]. It is worth mentioning that co-infection is the existence of a bacterial infection within the first 48 hours of the diagnosis of COVID-19, confirmed by culture of orotracheal secretion or blood cultures, molecular panels for viruses and bacteria and/or antigenic detection methods [2]. Superinfection or secondary infection is the appearance of a bacterial infection after 48 hours of admission, with at least one positive culture of a respiratory sample (endotracheal aspirate, bronchoalveolar lavage), blood culture, urine culture, or the result of molecular tests, such as multiplex PCR and, in a clinical context, consistent with infection [2]. Secondary infections are used to be considered healthcare-associated infections. The specific occurrence of bacterial superinfection in COVID-19 is unclear. Although it is estimated that 3-10% of patients may present it, a meta-analysis placed these figures at around 7% [2,25,26]. A randomized cohort found that 57% of patients with COVID-19 received antibiotics in the first 48 hours of hospital admission, and less than 4% were diagnosed with bacterial co-infection [27]. Another review determined that more than 72% of patients with COVID-19 received antimicrobial therapy, only 8% meriting it [28]. The high prescription of antibiotics occurred mainly during the first peak of the health emergency, with higher rates of ICU admission [29]. In addition, broader-spectrum antibiotics were used during the weeks with the highest number of cases [30]. The use of antibiotics promotes the growth and proliferation of bacterial microorganisms capable of resisting their antimicrobial properties, known as bacterial resistance [31]. Since the beginning of the pandemic, several investigations have reported an increase in multidrug resistance in bacterial and fungal infections [3,29]. It has been seen that the resistance of Staphylococcus aureus against clindamycin, erythromycin, and oxacillin is up to 90% [29]. One study found 53% resistance to erythromycin in Streptococcus pyogenes, 58% to tetracyclines, and co-resistance to both drugs in 40% [32]. The frequency of methicillin-resistant S. aureus has increased, and studies in patients with bacterial infections secondary to COVID-19 show methicillin resistance in all strains of coagulase-negative Staphylococcus and S. aureus [33]. Gram-negative bacteria have acquired higher rates of antimicrobial resistance, including Escherichia coli, Acinetobacter baumannii, Pseudomonas aeruginosa, and Klebsiella pneumoniae [34]. Resistance has been described in 76% and 91% to carbapenems in K. pneumoniae and A. baumannii, respectively [33]. A study in patients with COVID-19 showed Enterobacteriaceae resistance to cefepime at 43%, ceftazidime at 47%, piperacillin at >67%, and trimethoprim-sulfamethoxazole at 75%. All of them showed amikacin susceptibility [29]. There are already reports of acquired resistance to ceftolozane-tazobactam and ceftazidime-avibactam. This phenomenon is a major public health problem that claims thousands of lives annually worldwide [35], and it is estimated that by 2050 it could reach 10 million deaths [36].

Learned lessons

Safe antimicrobial stewardship strategies aim to limit indiscriminate use to decrease antimicrobial resistance [29]. The use of antibiotics in COVID-19 is not yet standardized, and there is no evidence for their routine use. Instead, factors have been proposed that determine infectious-bacterial processes that warrant its use (Table 1).

**Table 1:** Predictors of bacterial co-infection in SARS-CoV-2 infection.

Predictors of bacterial co-infection in COVID-19
Clinical features
New onset or exacerbation of fever
Changes in sputum characteristics
Progressive clinical deterioration
Laboratory test
Signs of organ failure
Leukocytosis
Neutrophilia and lymphocytopenia
Increased C-reactive protein
Increased levels of procalcitonin
Images
New lobular consolidation patterns in chest X-ray

Condition of the Patient

The clinical and hemodynamic condition of the patient is essential to suspect bacterial coinfection. For example, in patients with COVID-19, the presence of signs of systemic inflammatory response, dyspnea, persistent fever, tachycardia, and data of unexplained tissue hypoperfusion before day 7 of symptoms are useful to identify additional infectious processes [37]. Studies have shown increased sputum production in patients with bacterial coinfection [27]. In addition, a change in the characteristics of the sputum and the return or exacerbation of febrile episodes may also indicate the development of underlying bacterial pathology [38].

Lab Tests

The role of inflammatory biomarkers in favoring decision-making has been evaluated. One study found that electrolyte disturbances, anemia, hypoproteinemia, and signs of organ failure such as acute liver function impairment, acute kidney injury, troponin-mediated heart damage, and death were higher in the group of patients with high suspicion of bacterial pneumonia added to COVID-19 [37]. D-dimer levels increased in patients with COVID-19, being normal in bacterial pneumonia [38]. C-reactive protein (CRP) is used to guide the use of tocilizumab; however, bacterial superinfection must be ruled out. Nevertheless, studies defend the usefulness of CRP for initiating antibiotics since they found that patients with COVID-19 and coinfection had higher levels than controls [39,40]. Therefore, it is proposed to combine clinical and laboratory parameters to define the presence of bacterial coinfection. Leukocytosis is not an absolute criterion for the existence of bacterial coinfections, taking into account phenomena such as the demarginalization of leukocytes after the use of steroids. However, the authors state that in patients with confirmed coinfection, in the absence of steroid use, there is a higher leukocyte count [25,41]. Others refer that lymphocytosis is characteristic of viral pathologies and that a 25% increase in leukocytes is suggestive of the development of bacterial processes with a positive predictive value of 85.71% [38,41].

Usefulness of Procalcitonin

Despite the controversy over the usefulness of procalcitonin (PCT) in differentiating viral-bacterial etiologies [42], several publications show that elevated PCT is associated with the severity of SARS-CoV-2 infection. One study found that more than 70% of COVID-19 patients without co-infection at admission had PCT values ​​<0.25 ng/mL [43]. PCT has a high negative predictive value in the diagnosis of bacterial co-infection if it is <0.1 ng/ml [27]. When it is ≥0.1 ng/ml, it could be related to bacterial co-infection and greater severity in patients with COVID-19 [44]. A ferritin/procalcitonin (F/P) ratio >1250 is useful for differentiating bacterial pneumonia and COVID-19 (sensitivity: 78%, specificity: 59%, and area under the receiver operating characteristic [ROC] curve: 0.79 [OR: 4.9; CI: 1.5-16.1, p=0.009]) [45]. Likewise, higher ferritin concentrations have been seen in the group of patients with COVID-19 without bacterial co-infection and higher PCT levels in bacterial pneumonia [45]. Further studies are required to assess the accuracy and efficacy of this index.

Radiological Findings

The American College of Radiology recommends radiography as the initial image for patients with respiratory symptoms. CT is more sensitive, it allows to characterize the lesions, classifying the findings into opacities due to bacterial infection versus the typical ground glass opacities seen in COVID-19 [38,46]. There are atypical radiological findings that make us consider a different etiology, such as budding or subpleural tree nodules, cavitations, isolated or multilobe consolidation without ground glass, loculated pleural effusion, and mediastinal lymph adenomegaly [47,48]. The presence of new bilateral opacities or consolidation pattern were found in almost all patients with bacterial superinfection, and infrequent in COVID-19 without superinfection [38].

Proposed management

**Figure 3:**
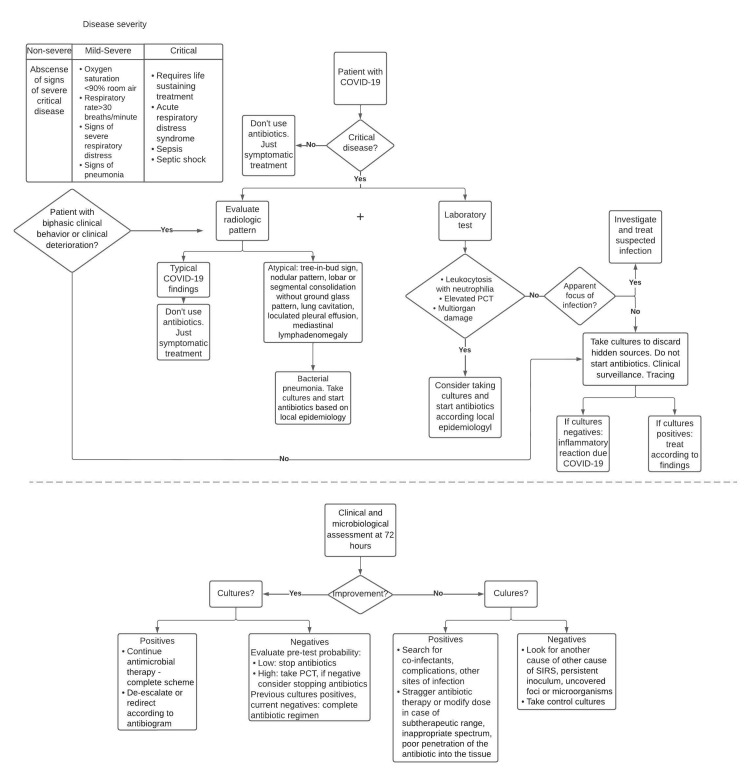
Proposed decision-making algorithm for the use of antibiotics in COVID-19.

It is suggested to classify the patient according to the severity of the disease based on clinical and laboratory test criteria. Patients with mild-to-moderate disease benefit from symptomatic management without antibiotics. Regardless of clinical classification, patients should be closely monitored. In critically ill patients, a useful tool is the calculation of sequential organ failure assessment (SOFA) and delta-SOFA, which determines the degree of organic dysfunction upon admission to the ICU and during its stay [49,50]. In patients with severe disease, it is necessary to perform a chest CT to assess the presence of typical radiological signs of COVID-19 or bacterial processes that indicate taking cultures and early initiation of antibiotic therapy. Laboratory test results showed elevated PCT, leukocytosis with neutrophilia, and evidence of objective multi-organ damage suggest bacterial infection, requiring serial cultures and antibiotic treatment according to epidemiology, antimicrobial susceptibility patterns, established management guidelines, and local guidelines of bacterial resistance; always choosing the antimicrobial with less collateral damage. If the suspicion of added infection persists without an established infectious focus, cultures should be taken to rule out hidden sources of infection. Initiation of antibiotics is not recommended until positive cultures are obtained. If the diagnosis of bacterial co-infection or superinfection was determined after 72 hours of clinical and microbiological surveillance, patients who show improvement and have positive cultures should continue the established antimicrobial regimen, discontinue antibiotics with a narrower spectrum, or redirect to another antimicrobial depending on the microbiological isolation obtained. In case of negative cultures and low pre-test probability, it is recommended to suspend antibiotics. If the pre-test probability is high, a new PCT measurement is recommended to define antibiotic suspension. When there is no improvement or there is clinical deterioration, obtaining negative cultures requires searching for another agent, microorganisms, or foci not covered with the current scheme. In addition, new cultures must be performed. In patients without clinical improvement with positive cultures, co-infections and other sites of infection should be sought while assessing the need to increase the dose of antibiotics used at the time or to stagger antimicrobial therapy if poor penetration is noted in the tissues of the drug used or an inappropriate spectrum in relation to the microorganism found in the culture. Finally, in patients with progressive clinical worsening or biphasic clinical behavior, a new radiological evaluation should be performed in search of imaging signs typical of COVID-19 or suggestive of bacterial pathology, in addition to taking cultures. The duration of antimicrobial treatment should be as short as possible (Figure 3).

## Conclusions

The health emergency has led to the indiscriminate use of antibiotics, leading to severe consequences such as an increase in allergies, costs to the health system, and the increase in resistant strains. To date, most of the research on COVID-19 has very low methodological quality, which makes it challenging to use and apply. There is no consensus to discriminate between SARS-CoV-2 infection and bacterial coinfection or superinfection due to their similarity in presentation. Clinical condition and laboratory tests are important predictors that can help discern when to start antibiotics considering the local antibiogram. Frequent evaluations should be made to determine the continuity or staggering use of the antibiotics.
